# Decision aids on breast conserving surgery for early stage breast cancer patients: a systematic review

**DOI:** 10.1186/s12911-020-01295-8

**Published:** 2020-10-22

**Authors:** Jing Si, Rong Guo, Xiang Lu, Chao Han, Li Xue, Dan Xing, Caiping Chen

**Affiliations:** 1 Department of Breast Disease, The First Hospital of Jiaxing and The First Affiliated Hospital of Jiaxing University, No. 1882, South Zhonghuan Rd., Jiaxing, 314000 China; 2 Department of Breast Surgery, Fudan University Shanghai Cancer Center, No. 270, Dongan Rd., Shanghai, 200032 China

**Keywords:** Breast cancer, Decision aids, Decision making, Decision support, Breast conserving surgery, Breast conserving therapy

## Abstract

**Background:**

Breast cancer is a worldwide health concern. For early stage breast cancer patients, choosing the surgical method after diagnosis is always a dilemma. Decision aids designed for use by patients are tools which may help with surgical decision making for these patients.

**Methods:**

We screened through MEDLINE, EMBASE, PubMed and Web of Science using the inclusion criteria which included (1) newly diagnosed patients with early stage breast cancer, (2) outcomes/results involving surgical options including breast conserving surgery. The search strategy used these key words or the combination of these words: “breast cancer”, “decision aid”, “decision making”, “decision support”, “breast conserving surgery”, “breast conserving therapy”.

**Results:**

A total of 621 studies were identified, but only seven studies were included. Results were synthesized into narrative format. Various patterns of decision aids designed for use by patients were implemented. Mostly were educational materials via booklet, video or CDROM with or without assistance from surgeons. After decision aids, four studies showed that patients were more likely to change their original choices into mastectomy or modified radical instead of sticking to breast conserving surgery. Other results such as knowledge of breast cancer and treatments, decisional conflict and satisfaction, psychological changes after surgery and quality of life were all showed with a better trend in patients with decision aids in most studies.

**Conclusion:**

Decision aids on breast conserving surgery made it easier for patient involvement in surgical decision making and improved decision-related outcomes in most early stage breast cancer patients. With more attention, improving procedures, and better interdisciplinary cooperation, more research is necessary for the improvement of decision aids. And we believe decision aids with agreed objective information are needed.

## Background

Breast cancer is the most common malignancy diagnosed in women [1, 2]. According to the latest statistics from American Cancer Society, approximately 13% of women (1 in 8) will be diagnosed with invasive breast cancer in their lifetime [3]. With improved detective methods and various treatments, more patients were diagnosed at early stages, which is an important predictor for better prognosis. For patients with early stage breast cancer, surgery is always part of the treatment. Several randomized control trials showed no difference in local recurrence rate, overall survival and quality of life among patients treated with breast conserving therapy, mastectomy and modified radical mastectomy [4, 5]. Thus, patients with early stage breast cancer should face the dilemma of choosing the surgical method after diagnosis.

In the past, treatment decisions were often made by surgeons with little patients’ involvement. While recently, instead of leading by surgeons, patients are willing to discuss with their surgeons and play a role in treatment decision making [6, 7]. Although most surgeons believed that patients were included during decision making, patients still felt incompetent to take part in the process of decision making, owe to the fact that they lack relevant information [7].

It is important to present the information about the choices patients need to make neutrally, to clarify their personal values and to express their preferences, to achieve the personalized treatments. Decision aids (DAs) designed for use by patients are tools which can promote the involvement of patients in decision making. These tools help patients make informed choices by telling the alternatives in detail, sharing the risks and benefits of each choice and recognizing personal values [7]. Unlike traditional health educational materials, DAs share specific information which is directly related to decision making with focus on patients’ personal values. It is a model that patients make decisions more effectively and responsibly together with their surgeons. It is a way, through which patients can feel higher degree of participation and communicate with surgeons more smoothly. Also, patients will have practical expectations of the treatment they may take. Thus, for patients with early stage breast cancer, DAs play a significant role in the treatment.

In this review, we focused on all kinds of decision aids designed for use by patients. Some of these decision aid tools are used only by patients, others are used in a shared pattern by both clinicians and patients. The objective of this systematic review is to examine research on decision aids that specifically targets breast conserving surgery, one of the surgical options for early stage breast cancer patients.

## Methods

### Sources and search strategy

This systematic review was conducted according to the principles of the PRISMA statement [8]. Four databases were searched for primary research studies: MEDLINE, EMBASE, PubMed and Web of Science. Studies were eligible if: (1) patients were newly diagnosed with early stage breast cancer; (2) Outcomes/results involving surgical options, including breast conserving surgery, were reported related to the use of a DA. A DA was defined as a tool which provided information about optional surgical method and relevant outcomes [9]. The format of DAs can be various, including video, audio, paper-based or multimedia. Articles were excluded if (1) they were not in English, (2) they were pilot studies, and (3) the full text of the study was not available. Keywords used to develop the search strategy comprised “breast cancer”, “decision aid”, “decision making”, “decision support”, “breast conserving surgery”, “breast conserving therapy”. The search strategy was designed to be maximally inclusive (see Appendix Table 2).

### Review selection process

**Fig. 1:**
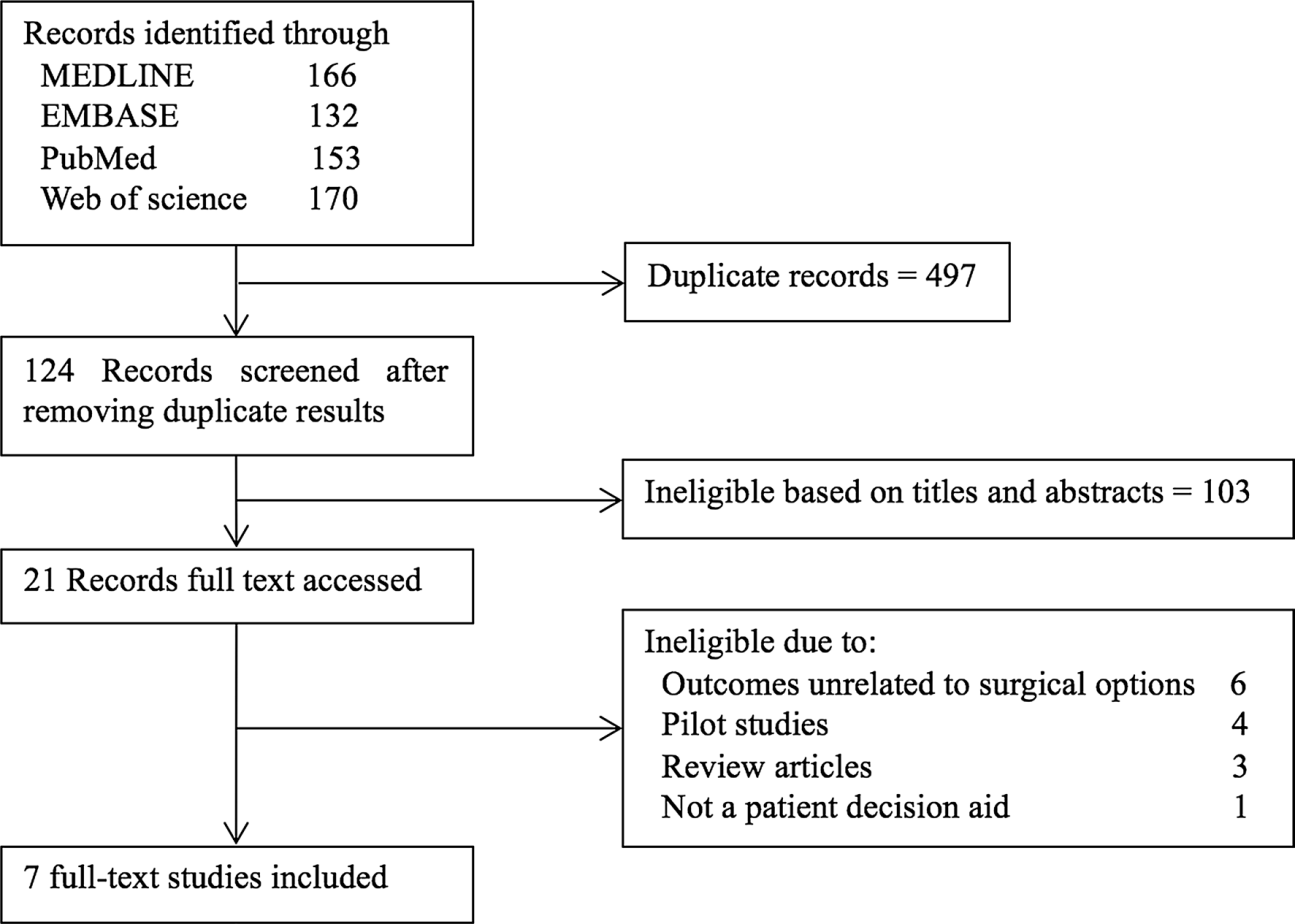
Systematic review flow diagram

The selection process of articles included in our systematic review was showed in Fig. 1. After removing duplicate results, we screened titles and abstracts to identify potentially eligible articles. The full text of these articles was reviewed to list articles met our inclusion criteria. Finally, seven studies were included [10–16]. A PRISMA diagram was showed in the “Appendix” (see Table 3). Quality and risk of bias were assessed at a study level using the QualSyst scoring system (see “Appendix” Table 4). These articles were showed in following elements in Table 1: authors, year of publication, design, sample, intervention, control, measurement tools, and outcomes.

**Table 1:** Overview of the articles

Authors	Year	Country	Design	Sample	Intervention	Control	Measurement tools	Outcomes	Qualsyst
Lam [11]	2013	China	Randomized control trial	276 patients with early stage BC DA: 138 patients Control: 138 patients	Take-home booklet	The standard information booklet	Treatment decision-making difficulties and decisional conflict scale, knowledge scale, decision regret, Hospital Anxiety and Depression Scale (HADS)-Anxiety subscale and HADS-Depression subscale, decision regret	Choice of surgery did not differ between the DA and control arms. (BCS, MRM or MRM + BR/MRM or MRM + BR) The DA group had lower decisional conflict scores 1 week after consultation (*P* < 0.016), lower decision regret scores 4 (*P* < 0.026) and 10 months (*P* < 0.014) after surgery and lower depression scores 10 months after surgery (*P* < 0.001)	0.89
Jibaja-Weiss [10]	2011	USA	Randomized control trial	76 patients with early stage BC (I–IIIA) DA: 40 patients Control: 36 patients	An entertainment-based decision aid for breast cancer treatment along with usual care	Usual care only	A questionnaire for evaluating breast cancer knowledge, Satisfaction with Decision Scale (SWD), Satisfaction with the Process of Making a Treatment Decision scale (SWDMP), low-literacy version of the Decisional Conflict Scale	Patients in DA group prefer to MRM (59.5% vs. 39.5%, *P* = 0.018) than BCS (40.5% vs. 50.0%). (BCS or MRM) DA group showed a significant improvement in knowledge at the pre-surgery assessment (*P* < 0.001). Both groups showed decreased decisional conflict over the assessment periods (*P* < 0.001)	0.57
Whelan [15]	2004	Canada	Randomized control trial	201 patients with stage I or II BC and 20 surgeons DA: 94 patients and 10 surgeons Control: 107 patients and 10 surgeons	Decision board (written and visual information) Takes 20 min	Usual consultation style without using the decision board	A 44-item questionnaire for patient knowledge, decisional conflict scale, effective decision-making subscale of the decisional conflict scale, the Spielberger State Anxiety Inventory and the Centre for Epidemiologic Studies Depression scale	Patients in DA group were more likely to choose BCS (94% vs. 76%, *P* = 0.03). (BCS or MT) The DA group had higher knowledge scores about their treatment options (66.9 vs. 58.7; *P* < 0.001), had less decisional conflict (1.40 vs. 1.62, *P* = 0.02), and were more satisfied with decision making (4.50 vs. 4.32, *P* = 0.05)	0.75
Street [13]	1995	USA	Randomized control trial	60 patients with stage I or II BC DA: 30 patients Control: 30 patients	Multimedia program (including text, graphic display, audio narration, music, and audio–video clips) Takes 30–45 min	An 8-page brochure, Care of Patients with Early Breast Cancer Takes 15–20 min	An 11-item, multiple choice test for knowledge about BC treatment, an 8-item instrument for patients’ optimism, behavioral and self-report measures for patient involvement and physician communication, modified Perceived Involvement in Care Scale (PICS), modified Perceived Decision Control (PDC), 5-item doctor facilitation subscale of PICS	More patients educated with the computer chose BCS (76%) than did those reading the brochure (58%). (BCS or MT) Patients using the computer program scored higher in the knowledge test (mean, 82.6%; SD, 11.58%) after the intervention than did patients reading the brochure (mean, 76.4%; SD, 13.77%). The only variable predicting a patient’s optimism was knowledge (r = 0.31, *P* < 0.01)	0.61
Wilkins [16]	2006	USA	Nonrandomized trial with concurrent control	101 patients with stage I or II BC DA: 52 patients Control: 49 patients	Educational video Takes 60 min	Written educational materials	Autonomy and Information-Seeking Preferences, Self-Efficacy to Communicate with Physician/Manage Disease, Knowledge about Breast Cancer, State-Trait Anxiety Inventory, Perceived Involvement in Care, Satisfaction with Decision	25% of people who saw the video chose mastectomy compared to 14% of those who did not see the video (*P* = 0.18; OR = 2.00, 95%CI 0.72–5.53). (BCS or MT) No statistically significant differences between the 2 groups measured with all the scales	0.82
Molenaar [12]	2001	the Netherlands	Nonrandomized trial with concurrent control	180 patients with stage I or II BC DA: 92 patients Control: 88 patients	Interactive Breast Cancer CDROM Takes 70 min	Standard care including oral information and brochures	A 4-item scale for satisfaction with the decision-making process, 3 out of 4 items of the “effective decision-making” subscale of the DCS for satisfaction with the decision, the MOS20 and the EORTC QLQ-BR23	No difference between the CDROM and standard care condition in the treatment decision made. Most patients in both conditions selected BCS (CDROM: 75%; standard care 68%). (BCS or MT) CDROM patients expressed more satisfaction with information, the decision-making process, and communication. CDROM patients reported better physical functioning, less pain and fewer arm symptoms	0.86
Whelan [14]	1999	Canada	Nonrandomized trial with historical control	patients with clinical stage I or II BC and 7 surgeons DA: 175 patients and 7 surgeons Control: 194 patients	The surgical Decision Board Takes 20 min	Before DA	A 6-point Likert scale for patient preference, questionnaire for general acceptability of the decision aid, a 14-statement response for patient comprehension, a 5-point Likert scale for patient satisfaction with information and decision-making	The rate of breast-conserving surgery decreased when the Decision Board was introduced (88% vs. 73%,* P* = 0.001). (BCS or MT) 98% patients using the Decision Board reported that the Decision Board was easy to understand, and 81% indicated that it helped them make decisions. Surgeons found the Decision Board to be helpful in presenting information to patients in 91% of consultations	0.64

*BC* breast cancer, *DA* decision aids, *BCS* breast-conserving surgery, *MRM* modified radical mastectomy, *BR* breast reconstruction, *MT* mastectomy

## Results

### Overview of studies

A total of 621 studies were identified, but only seven studies were included, among which four were conducted in the United States, three in Canada, one in the Netherlands and one in People’s Republic of China. Four out of seven articles were randomized control trials (RCTs), two were non-randomized trials with concurrent controls, and one was non-randomized trial with historical control. In three RCTs, patients were randomly assigned into two groups, which were intervention group and control group [10, 11, 13]. However, only one study explained the random assignment procedure clearly [11].

Most articles had inclusion and exclusion criteria in detail. Generally, eligible patients were newly diagnosed with early stage breast cancer and were suitable for either breast conserving surgery or mastectomy. However, the specific inclusive stage was different. Most articles were stage I–II, while two articles had stage III patients [10, 11]. The exclusion criteria were similar in these articles, such as non-malignant breast diseases, recurrent or metastatic breast cancer, poor health condition which could not tolerant surgical treatment, and mental disorder which could not cooperate during decision aids and measurements.

Few articles had organized special team to select candidates. Wilkins et al. [16] set up a team called the BCC (Breast Cancer Center) Tumor Board, which included 25 breast disease experts in several specialized fields, to confirm the acceptation in the trial.

The sample sizes ranged from 60 to 276. However, only three articles explained the intended sample sizes and the power analysis of the trials [11, 12, 15]. Moreover, during the trials, there were quite a lot of patients got excluded, due to losing follow-up, poor cooperating, and unfinished questionnaires. When analyzing patients’ options, more patients were excluded because they had not decided yet [11]. While, no article compared the baseline of these patients with finally inclusive ones.

### Intervention and control

Various patterns of decision aids were implemented in the intervention group, which led to the diversity of each corresponding control. For most articles, patients in the intervention group were given educational materials via booklet, video or CDROM without assistance from surgeons. They could discuss with their friends and family members during decision making. While in two articles, instruments were presented by trained surgeons during the consultation, and patients could discuss with their surgeons and raise questions [14, 15]. For patients in the control group, usual care and consultation were given. Some articles had brochure or written materials with similar information only in the written form [13, 16].

### Outcomes

As we can see in Table 1, the measurement tools were different in each study, ranging from scales with examined reliability and validity, such as Decisional Conflict Scale (DCS) and Hospital Anxiety and Depression Scale (HADS), to modified scales or self-made questionnaires.

#### Final surgical option

In these studies, overall preference on surgical treatment was similar. Patients were more likely to receive breast conserving surgery, which showed the same trend as the statistics on surgical treatment for early stage breast cancer patients in the National Cancer Data Base [17].

After decision aids, some patients changed their choices. Among these studies, four of which showed that patients with decision aids were more likely to change their original choices into mastectomy or modified radical mastectomy [14, 16]. While two studies had opposite results. Whelan et al. [15] found patients with decision aids were more likely to choose breast conserving surgery (94% vs. 76%, *P* = 0.03). Street et al. [13] found more patients chose breast conserving surgery in the intervention group than control group (76% vs. 58%), although the difference did not reach statistical value.

#### Knowledge of breast cancer and treatments

Most articles evaluated patients’ knowledge of breast cancer and treatment options [10, 11, 13, 15]. The measurement tools were various questionnaires. Some articles showed that patients with decision aids had better knowledge than control group after the introducing, while no difference in follow-up assessments [10, 13]. Whelan et al. [15] also found that decision aids group had higher knowledge scores (*P* < 0.001), especially knew better about the same survival rate in breast conserving surgery and mastectomy. However, one study showed no significant difference in knowledge after decision aids and consultation [11].

#### Decisional conflict and satisfaction

Decisional Conflict Scale (DCS) and the subscale of DCS were used for assessing patients’ decisional conflict and satisfaction with final decision or decision-making process. Satisfaction with Decision Scale (SWD) and Satisfaction with the Process of Making a Treatment Decision scale (SWDMP) were also used for assessing. Generally, patients in the intervention group had no less decisional conflict scores than the control group after consulting with surgeons [10, 11, 15]. Also, Lam et al. [11] found that, compared with patients in the intervention group, patients in the control group reported greater decision regret 4 months (*P* = 0.026) and 10 months (*P* = 0.014) after surgery. As for patients’ satisfaction, three articles showed no difference in two arms [10, 11, 16], while two articles found patients with decision aids had better satisfaction with final decision [12, 15].

#### Psychological changes after surgery

Many psychological scales were used, such as Hospital Anxiety and Depression Scale (HADS), the Spielberger State Anxiety Inventory, and the Centre for Epidemiologic Studies Depression scale. Most studies showed that patients’ anxiety level was lower after consultation and would decrease in the assessment after surgery. One article showed that 10 months after surgery, patients in the control group had higher HADS-Depression scores than the intervention group (*P* = 0.001), while the HADS-Anxiety scores did not differ between groups [11]. In addition, Street et al. [13] found that the only predictor of patients optimism was their knowledge of breast cancer and treatment options (*P* < 0.01). The more knowledge they got, the more optimistic they would be.

#### Quality of life

Unfortunately, few articles retrieved quality of life as outcome. Molenaar et al. [12] used MOS20 and EORTC QLQ-BR23 to measure the quality of life, reported that patients with decision aids had better general health, better physical functioning, less pain, and fewer arm symptoms.

## Discussion

The purpose of this systematic review was to determine information requirement of patients diagnosed with early stage breast cancer facing a surgical choice and the role played by decision aids in the treatment decision making process. Generally, the contents of decision aids included background of breast cancer, introduction of treatment options, review of benefits and risks of each option, and personal values clarification. This information could come from guidelines, recent researches, and surveys of surgeons and fellow patients. We found the final surgical option could be affected by decision aids. However, the influence was inconsistent. There were several explanations for this differentiation. First, two articles compared breast conserving surgery with modified radical mastectomy included patients with stage III breast cancer, who tended to choose mastectomy considering the possibility of recurrence. Second, with the development of breast reconstruction, patients would probably choose mastectomy due to the cosmetic thoughts and lack of radiotherapy. Third, Chinese patients usually had smaller breasts than western women, which could be one possible reason for decreased breast conserving surgery. Last but not least, there could be risk of bias that some decision aids encouraged patients to choose specific surgical option rather than other alternatives. Although this kind of bias was not unacceptable in decision aids as long as the knowledge in decision aids was true and objective, this could be one of the reasons why the influence of decision aids on surgical options was inconsistent. Other results such as knowledge of breast cancer and treatments, decisional conflict and satisfaction, psychological changes after surgery and quality of life were all showed with a better trend in the intervention group.

Also, there were several aspects with no analysis, while we believed is necessary. First, the feasibility and completion rate of decision aids were not assessed. Considering the difference in patients’ educational level and patterns of decision aids, the feedback of implementing decision aids could be different. Wilkins et al. [16] found that most patients with decision aids thought the information was easy to understand (80%), the length of decision aids was properly (65%), and the information presented was neither too little nor too much (86%). Similarly, Whelan et al. [14] showed that 98% patients in the intervention group thought the Decision Board was easy, and 81% patients expressed that decision aids were useful for treatment decision making. Jibaja-Weiss et al. [10] even innovated decision aids for patients with low health literacy, which was more personalized. Also, the pattern of decision aids was another factor influenced the feasibility and completion rate. Although we found that information presented in different forms, such as written, visual and oral, could all be helpful, studies compared different forms showed that decision aids with pictures were much clearer for patients than only the words [18, 19]. Second, the reliability and validity of those measurement tools were not tested, especially those modified scales and self-made questionnaires. Some modified scales were designed for specific kind of patients, which should be tested before using officially. We believe interdisciplinary cooperation with psychological department can help us more with the scales.

Generally, there are many factors which can affect surgical options, such as age, race, tumor characteristics, socioeconomic factors, genetic factors, and patients’ own perceptions [20]. And the goal of decision aids is to help patients find the true preference of treatment options. Thus, the factors influence decision aids may afterwards affect treatment decision making. Studies showed that surgeons’ recommendation and patients’ concerns about local recurrence or breast loss were the strongest factors which could influence treatment preference [14, 21]. In most situations, patients requested recommendation from their surgeons [14]. Compared with medical and radiation oncologist, patients were more likely to interact with surgeons (*P* = 0.05) and felt involved [13]. While, surgeons’ practice type, communication style, hospital factors and even gender were associated with surgical decision [20, 22, 23].

Decision aids have four-level goals [18]. First, decision aids should show patients the perception of having a choice. Whelan et al. [15] found that patients in the intervention group tended to perceive that they had a choice to make than patients in the control group (87% vs. 69%, *P* = 0.07). Also, there was a correlation between the degree of perception and satisfaction with the decision (*P* < 0.01) [24]. Second, patients should learn more information about breast cancer and treatment options via decision aids. Several studies showed better knowledge scores in the intervention group [10, 13, 15]. Third, decision aids should decrease the difficulties of treatment decision making. Most patients found decision aids useful in the study [14]. Jibaja-Weiss et al. [10] showed 10.5% patients in the control group were unsure about their surgical options, while all the patients in the intervention group had made their choices about the surgery, which implied the role of decision aids indirectly. Fourth, decision aids should finally improve patients’ quality of life. Molenaar et al. [12] measured it with scales, showed that decision aids could lead to better quality of life. This is always the final goal of decision aids.

There are some limitations to this systemic review. First, the lack of RCTs could contribute to selection bias. There were only four RCTs which were the top level of evidences, while only two of them clarified the specific procedures of randomization and proper sample sizes. Second, the heterogeneity of these articles was obvious, which could cause poor comparability. Samples, intervention methods, timing of decision aids and measurement tools listed in Table 1 were of great diversity, which would possibly decrease the reliability of meta-analysis. Third, quality of life was the final goal of decision aids, while few articles retrieved quality of life as outcome.

## Conclusion

Decision aids on breast conserving surgery play an important role in decision making regarding surgical options for early stage breast cancer. The surgical choices can be different after decision aids with more knowledge of breast cancer, less decisional conflict and better satisfaction with the final choice. For most patients, surgery procedure is complex, while pictures showing knowledge and prognosis outcome are clear and direct. Thus, we recommended visual decision aids. We believe that, with more attention, improving procedures, and better interdisciplinary cooperation, plenty of researches about decision aids will emerge, and decision aids with agreed objective information are needed.

## Data Availability

Data are available in a public, open access repository.

## Appendix

Search strategies for databases included in the review (see Table 2).

**Table 2:** Search strategies and records for databases (Searched in January 2019)

Database	Search strategies	Limits	Records
Medline	(Breast cancer and (decision aid or decision making or decision support) and (breast conserving surgery or breast conserving therapy)).mp.[mp = title, abstract, original title, name of substance word, subject heading word, keyword heading word, protocol supplementary concept word, rare disease supplementary concept word, unique identifier, synonyms]	Full text	166
		English language	
		Human	
Embase	(Breast cancer and (decision aid or decision making or decision support) and (breast conserving surgery or breast conserving therapy)).mp.[mp = title, abstract, original title, name of substance word, subject heading word, keyword heading word, protocol supplementary concept word, rare disease supplementary concept word, unique identifier, synonyms]	Full text	152
		English language	
		Human	
PubMed	("Breast Neoplasms"[Mesh] AND (decision aid[Title/Abstract] OR decision making[Title/Abstract] OR decision support[Title/Abstract]) AND (breast conserving surgery[Title/Abstract] OR breast conserving therapy[Title/Abstract])	Full text	153
		English language	
		Human	
Web of science	TS = (breast cancer) AND TS = ((decision aid OR decision making) OR decision support) AND TS = (breast conserving surgery OR breast conserving therapy)	Full text	170
		English language	

PRISMA diagram checklist (see Table 3).

**Table 3:** PRISMA diagram checklist

Section/topic	#	Checklist item	Reported on page #
Title			
Title	1	Identify the report as a systematic review, meta-analysis, or both	Title
Abstract			
Structured summary	2	Provide a structured summary including, as applicable: background; objectives; data sources; study eligibility criteria, participants, and interventions; study appraisal and synthesis methods; results; limitations; conclusions and implications of key findings; systematic review registration number	Abstract and key words
Introduction			
Rationale	3	Describe the rationale for the review in the context of what is already known	Background, paragraph 1–3
Objectives	4	Provide an explicit statement of questions being addressed with reference to participants, interventions, comparisons, outcomes, and study design (PICOS)	Background, paragraph 4
Methods			
Protocol and registration	5	Indicate if a review protocol exists, if and where it can be accessed (e.g., Web address), and, if available, provide registration information including registration number	N/A
Eligibility criteria	6	Specify study characteristics (e.g., PICOS, length of follow-up) and report characteristics (e.g., years considered, language, publication status) used as criteria for eligibility, giving rationale	Methods, paragraph 1
Information sources	7	Describe all information sources (e.g., databases with dates of coverage, contact with study authors to identify additional studies) in the search and date last searched	Methods, paragraph 1 and Table 2
Search	8	Present full electronic search strategy for at least one database, including any limits used, such that it could be repeated	Methods, paragraph 1 and Table 2
Study selection	9	State the process for selecting studies (i.e., screening, eligibility, included in systematic review, and, if applicable, included in the meta-analysis)	Methods, paragraph 2 and Fig. 1
Data collection process	10	Describe method of data extraction from reports (e.g., piloted forms, independently, in duplicate) and any processes for obtaining and confirming data from investigators	Methods, paragraph 2 and Fig. 1
Data items	11	List and define all variables for which data were sought (e.g., PICOS, funding sources) and any assumptions and simplifications made	Methods, paragraph 2 and Table 1
Risk of bias in individual studies	12	Describe methods used for assessing risk of bias of individual studies (including specification of whether this was done at the study or outcome level), and how this information is to be used in any data synthesis	Methods, paragraph 2 and Table 3
Summary measures	13	State the principal summary measures (e.g., risk ratio, difference in means)	N/A
Synthesis of results	14	Describe the methods of handling data and combining results of studies, if done, including measures of consistency (e.g., I2) for each meta-analysis	N/A
Risk of bias across studies	15	Specify any assessment of risk of bias that may affect the cumulative evidence (e.g., publication bias, selective reporting within studies)	Methods, paragraph 2 and Table 3
Additional analyses	16	Describe methods of additional analyses (e.g., sensitivity or subgroup analyses, meta-regression), if done, indicating which were pre-specified	N/A
Results			
Study selection	17	Give numbers of studies screened, assessed for eligibility, and included in the review, with reasons for exclusions at each stage, ideally with a flow diagram	Methods, paragraph 2, Results, paragraph 1 and Fig. 1
Study characteristics	18	For each study, present characteristics for which data were extracted (e.g., study size, PICOS, follow-up period) and provide the citations	Results and Table 1
Risk of bias within studies	19	Present data on risk of bias of each study and, if available, any outcome level assessment (see item 12)	Table 3
Results of individual studies	20	For all outcomes considered (benefits or harms), present, for each study: (a) simple summary data for each intervention group (b) effect estimates and confidence intervals, ideally with a forest plot	Results, outcomes and Table 1
Synthesis of results	21	Present results of each meta-analysis done, including confidence intervals and measures of consistency	N/A
Risk of bias across studies	22	Present results of any assessment of risk of bias across studies (see Item 15)	Table 3
Additional analysis	23	Give results of additional analyses, if done (e.g., sensitivity or subgroup analyses, meta-regression [see Item 16])	N/A
Discussion			
Summary of evidence	24	Summarize the main findings including the strength of evidence for each main outcome; consider their relevance to key groups (e.g., healthcare providers, users, and policy makers)	Discussion, paragraph 1
Limitations	25	Discuss limitations at study and outcome level (e.g., risk of bias), and at review-level (e.g., incomplete retrieval of identified research, reporting bias)	Discussion, paragraph 5
Conclusions	26	Provide a general interpretation of the results in the context of other evidence, and implications for future research	Conclusion
Funding			
Funding	27	Describe sources of funding for the systematic review and other support (e.g., supply of data); role of funders for the systematic review	Declarations

From: Moher et al. [25]

For more information, visit: www.prisma-statement.org

Qualsyst scores of studies included in the review (see Table 4).

**Table 4:** QualSyst scores of studies included in the review

References	1. Question sufficiently described	2. Design evident and appropriate	3. Subject selection method	4. Subject characteristics described	5. If randomized, was procedure reported	6. If blinded to investigators, was it reported	7. If blinded to subjects, was it reported	8. Outcome well defined and robust to measurement/misclassification bias	9. Sample size appropriate	10. Analysis described and appropriate	11. Some estimate of variance reported	12. Controlled for confounding	13. Results reported in sufficient detail	14. Results support conclusions	Total
[11]	2	2	1	2	2	2	0	2	2	2	2	2	2	2	0.89
[14]	1	2	1	1	2	0	0	1	2	2	0	1	2	1	0.57
[15]	1	2	2	1	2	0	0	2	2	2	2	1	2	2	0.75
[16]	2	2	2	2	1	0	0	2	1	1	0	0	2	2	0.61
[17]	2	1	2	1	N/A	N/A	N/A	1	2	2	2	1	2	2	0.82
[18]	2	1	2	2	N/A	N/A	N/A	1	2	2	2	1	2	2	0.86
[19]	2	1	2	1	N/A	N/A	N/A	0	2	2	0	1	1	2	0.64

## Abbreviations

BC: Breast cancer
DA: Decision aids
BCS: Breast-conserving surgery
MRM: Modified radical mastectomy
BR: Breast reconstruction
MT: Mastectomy
RCTs: Randomized control trials
BCC: Breast cancer center
DCS: Decisional conflict scale
HADS: Hospital anxiety and depression scale
SWD: Satisfaction with decision scale
SWDMP: Satisfaction with the process of making a treatment decision scale
PICS: Perceived involvement in care scale
PDC: Perceived decision control

## References

[1] Ferlay J, Colombet M, Soerjomataram I, Dyba T, Randi G, Bettio M (2018). Cancer incidence and mortality patterns in Europe: estimates for 40 countries and 25 major cancers in 2018. Eur J Cancer.

[2] Chen W, Zheng R, Baade PD, Zhang S, Zeng H, Bray F (2016). Cancer statistics in China, 2015. CA Cancer J Clin.

[3] DeSantis CE, Ma J, Gaudet MM, Newman LA, Miller KD, Goding Sauer A (2019). Breast cancer statistics, 2019. CA Cancer J Clin.

[4] Fisher B, Anderson S, Bryant J, Margolese RG, Deutsch M, Fisher ER (2002). Twenty-year follow-up of a randomized trial comparing total mastectomy, lumpectomy, and lumpectomy plus irradiation for the treatment of invasive breast cancer. N Engl J Med.

[5] Litiere S, Werutsky G, Fentiman IS, Rutgers E, Christiaens MR, Van Limbergen E (2012). Breast conserving therapy versus mastectomy for stage I–II breast cancer: 20 year follow-up of the EORTC 10801 phase 3 randomised trial. Lancet Oncol.

[6] Belkora JK, Miller MF, Dougherty K, Gayer C, Golant M, Buzaglo JS (2015). The need for decision and communication aids: a survey of breast cancer survivors. J Commun Support Oncol.

[7] Stacey D, Legare F, Lewis K, Barry MJ, Bennett CL, Eden KB (2017). Decision aids for people facing health treatment or screening decisions. Cochrane Database Syst Rev.

[8] Moher D, Shamseer L, Clarke M, Ghersi D, Liberati A, Petticrew M (2015). Preferred reporting items for systematic review and meta-analysis protocols (PRISMA-P) 2015 statement. Syst Rev.

[9] Elwyn G, O'Connor A, Stacey D, Volk R, Edwards A, Coulter A (2006). Developing a quality criteria framework for patient decision aids: online international Delphi consensus process. BMJ.

[10] Jibaja-Weiss ML, Volk RJ, Granchi TS, Neff NE, Robinson EK, Spann SJ (2011). Entertainment education for breast cancer surgery decisions: a randomized trial among patients with low health literacy. Patient Educ Couns.

[11] Lam WW, Chan M, Or A, Kwong A, Suen D, Fielding R (2013). Reducing treatment decision conflict difficulties in breast cancer surgery: a randomized controlled trial. J Clin Oncol.

[12] Molenaar S, Sprangers MA, Rutgers EJ, Luiten EJ, Mulder J, Bossuyt PM (2001). Decision support for patients with early-stage breast cancer: effects of an interactive breast cancer CDROM on treatment decision, satisfaction, and quality of life. J Clin Oncol.

[13] Street RL, Voigt B, Geyer C, Manning T, Swanson GP (1995). Increasing patient involvement in choosing treatment for early breast cancer. Cancer.

[14] Whelan T, Levine M, Gafni A, Sanders K, Willan A, Mirsky D (1999). Mastectomy or lumpectomy? Helping women make informed choices. J Clin Oncol.

[15] Whelan T, Levine M, Willan A, Gafni A, Sanders K, Mirsky D (2004). Effect of a decision aid on knowledge and treatment decision making for breast cancer surgery: a randomized trial. JAMA.

[16] Wilkins EG, Lowery JC, Copeland LA, Goldfarb SL, Wren PA, Janz NK (2006). Impact of an educational video on patient decision making in early breast cancer treatment. Med Decis Making.

[17] Kummerow KL, Du L, Penson DF, Shyr Y, Hooks MA (2015). Nationwide trends in mastectomy for early-stage breast cancer. JAMA Surg.

[18] Au AH, Lam WW, Chan MC, Or AY, Kwong A, Suen D (2011). Development and pilot-testing of a Decision Aid for use among Chinese women facing breast cancer surgery. Health Expect.

[19] Durand MA, Alam S, Grande SW, Elwyn G (2016). 'Much clearer with pictures': using community-based participatory research to design and test a Picture Option Grid for underserved patients with breast cancer. BMJ Open.

[20] Mac Bride MB, Neal L, Dilaveri CA, Sandhu NP, Hieken TJ, Ghosh K (2013). Factors associated with surgical decision making in women with early-stage breast cancer: a literature review. J Womens Health (Larchmt).

[21] Molenaar S, Oort F, Sprangers M, Rutgers E, Luiten E, Mulder J (2004). Predictors of patients' choices for breast-conserving therapy or mastectomy: a prospective study. Br J Cancer.

[22] Goel V, Sawka CA, Thiel EC, Gort EH, O'Connor AM (2001). Randomized trial of a patient decision aid for choice of surgical treatment for breast cancer. Med Decis Making.

[23] Mandelblatt JS, Berg CD, Meropol NJ, Edge SB, Gold K, Hwang YT (2001). Measuring and predicting surgeons' practice styles for breast cancer treatment in older women. Med Care.

[24] Janz NK, Wren PA, Copeland LA, Lowery JC, Goldfarb SL, Wilkins EG (2004). Patient-physician concordance: preferences, perceptions, and factors influencing the breast cancer surgical decision. J Clin Oncol.

[25] Moher D, Liberati A, Tetzlaff J, Altman DG, The PRISMA Group (2009). Preferred reporting items for systematic reviews and meta-analyses: the PRISMA statement. PLoS Med.

